# Sick of Feeling Sick: Management of Opioid Treatment-Induced Refractory Nausea Resulting in Suicidal Ideations With a Plan and Intent

**DOI:** 10.7759/cureus.60855

**Published:** 2024-05-22

**Authors:** Nkechinyere M Harry, Nnenna Okafor, Ibrahim Folorunsho, Ari Gao, Gibson O Anugwom

**Affiliations:** 1 Psychiatry, Vinnytsia National Pirogov Medical University, Vinnytsia, UKR; 2 Psychiatry, All Saints University, College of Medicine, Roseau, DMA; 3 Psychiatry, Badr Al Janoub Hospital, Najran, SAU; 4 Psychiatry, Menninger Department of Psychiatry and Behavioral Sciences, Baylor College of Medicine, Houston, USA

**Keywords:** scopolamine, suicide, nausea, methadone, opioids

## Abstract

In this case report, we present the case of a 60-year-old Caucasian male with a history of depression, anxiety, opioid dependence, and idiopathic polyneuropathy, admitted to an inpatient psychiatric unit for suicidal ideation. The patient's symptoms were characterized by months of intractable nausea, severe anxiety, suicidal ideation (SI), and significant unintentional weight loss in the context of methadone-assisted treatment. Over nine days in the hospital, a treatment strategy was developed and refined, which eventually achieved sustained relief from nausea and significant improvement in anxiety. The most effective pharmacological interventions included mirtazapine, scopolamine, and gabapentin.

## Introduction

Methadone, a long-acting opioid agonist, is an important medication in the management of chronic pain and opioid dependence, but its use requires careful consideration due to its complex pharmacokinetics and potential for serious side effects [1-2]. While it is generally manageable with standard antiemetic therapies, refractory nausea secondary to opioid agonists such as methadone can pose significant challenges to patient management and compliance [1-3].

We present a case of a 60-year-old Caucasian male with a history of depression, anxiety, and opioid dependence who presented with severe refractory nausea after initiation of methadone that persisted despite the administration of multiple antiemetic medications. This presentation was complicated by worsening depression, anxiety, and suicidal ideation (SI) with a plan to jump from a tall building due to the intensity of the nausea. 

This case report underscores the inherent challenges of managing a patient who is simultaneously dealing with opioid use and co-occurring psychiatric disorders. Furthermore, it serves to remind our readers of the importance of a comprehensive approach in such complex contexts. Addressing chronic pain, psychiatric well-being, and substance dependence requires a holistic treatment strategy. Here, the intractable nausea deepened the complexity of the patient's condition, intensifying both his physical discomfort and psychological turmoil.

## Case presentation

The patient was a 60-year-old Caucasian male with a history of laparoscopic hernia repair, Nissen fundoplication, opioid dependence, alcohol use disorder (in remission), depression, anxiety, and idiopathic polyneuropathy.

The patient developed pain as a result of lumbar spine bone spurs that began 11 years prior to his current presentation. He complained of severe pain in his lower spine that radiated to his bilateral lower extremities with associated neuropathy without motor deficits or incontinence. He underwent a spinal laminectomy, which unfortunately worsened his pain. Further workup, including labs and imaging tests such as computer tomography (CT) and magnetic resonance imaging (MRI) of the lumbar spine, did not yield a definitive diagnosis for his pain. The patient ultimately received a diagnosis of idiopathic polyneuropathy, a diagnosis of exclusion characterized by pain and dysfunction in multiple peripheral nerves.

He subsequently developed chronic pain, which was managed with a series of analgesics and other interventions. He first used tramadol for five years but became dependent on it, procuring it illegally through online sources and Mexico and taking up to 600 milligrams (mg) daily, far above his prescribed dose. After discontinuing tramadol three years ago, he was tried on oral non-steroidal anti-inflammatory drugs (NSAIDs), oral and injectable corticosteroids, and physical therapy, which were all ineffective. 

His most effective intervention for pain to date is a combination of a spinal cord stimulator and an oral opioid, initially hydrocodone. He was also concurrently managed with duloxetine (Cymbalta) for mood and neuropathic pain during this period but discontinued both due to blood pressure instability. A subsequent switch to buprenorphine was also unsuccessful due to orthostatic hypotension, and he was finally cross-tapered to methadone 10 mg three times daily (TID) six months before presentation. The patient reported that this regimen, combined with his spinal stimulator, was effective in managing his pain.

Unfortunately, the patient also developed unremitting and intractable nausea six weeks after initiating methadone, leading to poor oral intake and an unintentional weight loss of 50 pounds. The nausea was not associated with vertigo or food intake, although he did note some brief morning dysequilibrium on standing. It worsened on waking up, was constant throughout the day, and was mildly improved at night. The nausea persisted from the time of onset up to his presentation to our facility and was refractory to trials of metoclopramide, promethazine, ondansetron, and olanzapine. His nausea and associated functional decline worsened his depression and anxiety. His symptoms included a depressed mood, anhedonia, guilt regarding opioid dependence, insomnia, hopelessness, helplessness, and panic attacks. His outpatient psychiatrist started him on sertraline 50 mg daily, hydroxyzine 50 mg TID as needed (PRN), and eszopiclone 3 mg nightly (QHS). However, his depression and anxiety symptoms persisted, and he developed SI with a plan to jump off a tall building. After informing his outpatient psychiatrist of these developments, he was referred to our facility for an emergency psychiatric evaluation. On presentation, the patient scored 25 points out of a possible 30 points on the patient health questionnaire (PHQ-9), indicating severe depression, and again endorsed the aforementioned SI with a plan. He was voluntarily admitted to our inpatient psychiatric unit for stabilization and medication optimization. 

Pre-admission medications included sertraline 50 mg daily for depression and anxiety, eszopiclone 3 mg QHS for insomnia, pregabalin 200 mg TID for neuropathy and off-label for anxiety, methadone 10 mg TID for pain control, olanzapine 2.5 mg every day before noon (QAM) and every day at noon (QNOON) and 7.5 mg QHS for nausea, lisinopril 10 mg daily for hypertension, and rosuvastatin 40 mg daily for hypercholesterolemia.

Hospital course

The initial treatment plan was to reduce the dose of the offending agent, which was felt to most likely be methadone. However, the patient consistently declined, citing his long-standing pain, which, if unmanaged, would further worsen his distress. Our strategy therefore shifted to treating his nausea and psychiatric illnesses while continuing methadone (10 mg TID) and attempting to eliminate other potential causes of nausea. The patient complained of nausea but denied emesis throughout his illness and was able to take oral medications without difficulty. 

On day one, the patient's initial PHQ-9 score was 25/30, with self-reported depression and anxiety levels at 10/10. Due to nausea, his treatment with sertraline was stopped, and he was started on mirtazapine 7.5 mg at bedtime for sleep, depression, and anxiety. Pregabalin was unavailable in our facility, so gabapentin 300 mg three times daily was prescribed instead. Olanzapine was discontinued because it was ineffective, and meclizine 25 mg three times daily was introduced for antiemesis. The patient also received ondansetron 8 mg thrice daily for nausea and reported SI. He slept for only 1.5 hours at night due to anxiety, and he ate less than 25% of his lunch and less than 50% of his dinner.

On day two, the patient rated his depression at 9/10, and his anxiety remained at 10/10. He experienced temporary relief from nausea after each dose of meclizine, but the effect lasted only two to three hours before the nausea returned to its original severity. Consequently, meclizine was deemed to have limited efficacy and was discontinued. A scopolamine 1.5 mg patch was then applied every 72 hours. The patient still endorsed SI, slept for 3.7 hours, ate less than 50% of his lunch, and consumed 50% of his dinner.

By day three, the patient's depression decreased to 7/10 and her anxiety to 9/10. He reported a sustained resolution of nausea starting three hours after the scopolamine patch was applied. He did not exhibit anticholinergic symptoms such as dry mouth or constipation. The dosage of mirtazapine was increased to 15 mg at bedtime to further assist with appetite, depression, and anxiety. He denied SI and slept for 5.5 hours. His food intake improved to 25% for breakfast, 50% for lunch, and 75% for dinner.

On day four, the patient's depression score improved to 5/10, but his anxiety remained high at 9/10, especially in anticipation of replacing the scopolamine patch the following day. He reported no nausea, denied SI, slept for four hours, and ate 75% of his breakfast and lunch and 100% of his dinner.

Day five saw further improvement, with the patient's PHQ-9 score dropping to 13/30 after the scopolamine patch was replaced. He did not experience recurrent nausea, and the gabapentin dosage was increased to 600 mg three times daily to better manage his anxiety. He denied SI, slept for six hours, and fully consumed all his meals.

On day six, the patient rated his depression at 5/10 and his anxiety at 6/10. He received a single dose of oral lorazepam (1 mg) for anxiety, and mirtazapine was increased to 30 mg at bedtime. He denied SI, slept for 6.5 hours, and ate all meals completely.

Day seven showed a slight improvement in depression at 4/10 but increased anxiety at 8/10, likely due to concerns about potential discharge and relapse of nausea at home. The patient received lorazepam (1 mg) twice and began individual psychotherapy for coping skills. He denied SI, maintained a 6.5-hour sleep schedule, and consumed all meals fully.

On day eight, the patient's PHQ-9 score improved further to 7/30. After replacing the scopolamine patch, he noted no recurrent nausea and reported a significant reduction in anxiety to 4/10. He continued psychotherapy, denied SI, slept for seven hours, and ate all meals. He felt nearly ready for discharge.

By day nine, the patient's PHQ-9 score had improved remarkably to 3/30, with depression and anxiety levels at 2/10 and 3/10, respectively. He reported no nausea, denied SI, and was future-oriented. He actively participated in both individual and group therapies without needing lorazepam. A safety plan was completed with the patient and his wife, and a follow-up appointment was scheduled with an outpatient psychiatrist. The patient was discharged home with a sufficient supply of medications to last until his follow-up.

Medications at discharge 

Gabapentin 600 mg TID and 600 mg daily PRN for anxiety; mirtazapine 30 mg QHS for sleep, anxiety, and depression; scopolamine 1.5 mg patch every 72 hours (q72h) for nausea; ondansetron 8 mg TID PRN for breakthrough nausea; rosuvastatin 40 mg daily for hypercholesterolemia; lisinopril 10 mg daily for hypertension; and the naloxone (Narcan) kit were prescribed to the patient at discharge.

A summary of the patient's hospital course is presented in Table 1.

**Table 1:** A summary of the hospital course PRN: as needed; PHQ-9: patient health questionnaire; %: percent; tid: three times daily; mg: milligram; qhs: every night; <: lesser than; >: greater than; q72h: every 72 hours; PO: per oral

Day	Scheduled medications and treatments	PRN medications	Nausea	Suicidal ideation	Self-rated depression	Self-rated anxiety	PHQ-9	Sleep	% Meals eaten
1	Olanzapine and sertraline discontinued; started meclizine 25 mg TID; mirtazapine 7.5 mg QHS; gabapentin 300 mg TID	Ondansetron 8 mg three times for nausea	+	+	10-Oct	10-Oct	25/30	1.5 hours	Breakfast: 0%; Lunch: <25%; Dinner: <50%
2	Meclizine 25 mg discontinued; scopolamine 1.5 mg patch every 72 hours (q72h) started; mirtazapine 7.5 mg QHS; gabapentin 300 mg TID	None	+	+	10-Sep	10-Oct	-	3.7 hours	Breakfast: 0%; Lunch: <50%; Dinner: 50%
3	Scopolamine 1.5 mg patch; increased mirtazapine 15 mg QHS; gabapentin 300 mg TID	None	-	-	10-Jul	10-Sep	-	5.5 hours	Breakfast: 25%; Lunch: 50%; Dinner: 75%
4	Scopolamine 1.5 mg patch; mirtazapine 15 mg QHS; gabapentin 300 mg TID	None	-	-	10-May	10-Sep	-	4 hours	Breakfast: 75%; Lunch: 75%; Dinner: 100%
5	Scopolamine 1.5 mg patch replaced; mirtazapine 15 mg QHS; increased gabapentin 600 mg TID	None	-	-	-	-	13/30	6 hours	Breakfast: 100%; Lunch: 100%; Dinner: 100%
6	Scopolamine 1.5 mg patch; increased mirtazapine 30 mg QHS; gabapentin 600 mg TID	Lorazepam 1 mg PO once for anxiety	-	-	10-May	10-Jun	-	6.5 hours	Breakfast: 100%; Lunch: 100%; Dinner: 100%
7	Scopolamine 1.5 mg patch; mirtazapine 30 mg QHS; gabapentin 600 mg TID; plus individual psychotherapy	Lorazepam 1 mg PO twice for anxiety	-	-	10-Apr	10-Aug	-	6.5 hours	Breakfast: 100%; Lunch: 100%; Dinner: 100%
8	Scopolamine 1.5 mg patch replaced; mirtazapine 30 mg QHS; gabapentin 600 mg TID; plus individual psychotherapy	None	-	-	10-Mar	10-Apr	30-Jul	7.0 hours	Breakfast: 100%; Lunch: 100%; Dinner: 100%
9	Scopolamine 1.5 mg patch, gabapentin 600 mg morning and noon; plus Individual psychotherapy, then discharged home	None	-	-	10-Feb	10-Mar	30-Mar	-	Breakfast: 100%; Lunch: 100%

## Discussion

Nausea typically involves one of three pathways: direct stimulation of the chemoreceptor trigger zone (CTZ), reduction of gastrointestinal (GI) motility, and hypersensitization of the vestibular system in the inner ear, leading to nausea and sometimes vertigo. The treatment of nausea similarly consists of two categories: anti-serotonergic and dopamine receptor type 2 antagonists (promethazine, metoclopramide, olanzapine, and ondansetron) that act on both the CTZ and GI tract, and central-acting anticholinergic agents (scopolamine and meclizine) that primarily blunt vestibular sensitivity. Opioid-induced nausea may involve any of these three pathways or a combination thereof and may present after initiation of the opioid, during maintenance treatment, or upon discontinuation as part of a withdrawal syndrome. Identifying the causal factor of nausea and selecting a treatment for it is therefore complicated by its ubiquity and non-specificity and may require a detailed clinical assessment and multiple trials of agents or combinations thereof to achieve symptom relief [4]. 

Several elements of this patient’s clinical history point to strong vestibular involvement instead of the other two pathways. His nausea was not associated with food, and he denied constipation, reducing the likelihood of impaired GI motility. He did not respond to promethazine and metoclopramide, strong prokinetic agents that are known to improve nausea, where GI motility is implicated by blocking the D2 receptors. On the other hand, his non-response to ondansetron and olanzapine suggests that CTZ stimulation also did not play a determining role. Indeed, olanzapine is known to be highly effective in treating other medication-refractory CTZ-mediated emesis syndromes, including chemotherapy-induced vomiting [5]. The symptoms the patient did have were primarily motion-related. Although this patient denied frank vertigo, he endorsed postural disequilibrium and worsened nausea upon increased activity in the morning and improvement on going to bed, where motion is reduced. His partial response to meclizine and sustained response to scopolamine further support the involvement of the vestibular system, which is also implicated in motion sickness (and similarly treated). Interestingly, the superior efficacy of scopolamine over meclizine in motion-related nausea was reported in articles by Spinks et al. (2011) and Dornhoffer et al. (2004) [6-7]. In the randomized control trial (RCT) by Dornhoffer et al. (2004), where study participants received oral scopolamine, oral meclizine, or a sugar pill (placebo), scopolamine outperformed meclizine and placebo in controlling nausea associated with motion sickness [7].

We also observed that the patient did not experience nausea with prior opioids (tramadol, hydrocodone, and buprenorphine) but did with methadone, which raises questions about the differential effects of these opioids on various receptor systems and physiological pathways. Comparisons of rates of nausea and vomiting among opioids are sparse in the literature; a systematic review and meta-analysis by Dinges et al. (2019) concluded that among opioids at equianalgesic doses, only buprenorphine was associated with significantly higher rates of nausea and vomiting. Interestingly, our patient had no nausea or vomiting with buprenorphine. Neither methadone nor tramadol, the other opioids our patient was treated with, were shown to significantly increase nausea and vomiting in this review [8]. 

Our patient’s idiosyncratic side effect profile in response to opioids is consistent with the clinical observations of other providers. In a 2022 UpToDate article titled “Prevention and management of side effects in patients receiving opioids for chronic pain," Portenoy et al. report that in their clinical observations, one patient may experience intense nausea with one opioid medication and no nausea while receiving a different opioid [9]. No research study has elucidated the reason for this variation. Hence, we speculate that this phenomenon could be attributed to variations in opioid receptor binding affinities, interactions with other neurotransmitter systems, and/or potential contributions from individual genetic differences. This phenomenon underscores the need for further research into the specific mechanisms driving these differences and the potential for predictive markers of opioid side effect susceptibility.

## Conclusions

The patient's treatment outcome emphasizes the importance of considering the variability in opioid-induced side effects in patients with chronic pain and comorbid psychiatric disorders. The mechanisms for such variability remain unelucidated, and a holistic treatment approach, considering the psychiatric and somatic effects of psychoactive, opioid, and non-psychoactive medications, is most likely to succeed. Further studies to better understand the variables responsible for disparate opioid side effects will help guide prescribing practices and management of these side effects and ultimately improve patient outcomes. 
